# Neonatal intraoperative hypotension: individualizing BP while looking beyond pressure

**DOI:** 10.1016/j.jped.2026.101631

**Published:** 2026-10-15

**Authors:** Stephen J. Gleich

**Affiliations:** Mayo Clinic, Department of Anesthesiology and Perioperative Medicine, Rochester, Minnesota, USA

Blood pressure (BP) is among the most frequently measured physiologic variables during anesthesia, yet it remains an imperfect surrogate for the outcome clinicians actually seek to preserve: adequate blood flow and oxygen delivery to vital organs. This problem is particularly challenging in neonates, for whom gestational age, weight, underlying disease, anesthetic exposure, and surgical stress may all influence BP and the relationship between pressure and organ perfusion. Consequently, neonatal intraoperative hypotension (IOH) is easy to detect numerically but difficult to define physiologically.

In this issue of Jornal de Pediatria, Zhao et al. address this difficult and clinically important topic in a retrospective cohort of 1712 neonates undergoing non-cardiac surgery [1]. The authors defined IOH as a greater than 20% reduction in mean arterial pressure (MAP) from the individual preoperative baseline lasting more than 5 min. Using this definition, IOH occurred in 59.8% of neonates and was associated with prolonged postoperative recovery and greater rates of respiratory complications, acute kidney injury, and intraventricular hemorrhage. The authors should be commended for studying a large neonatal cohort and for moving away from a single arbitrary BP threshold based solely on age or weight.

The difficulty begins with the absence of a universally accepted definition of IOH in children. In a survey of pediatric anesthesiologists, most respondents considered a 20–30% reduction from baseline systolic BP to represent significant IOH, but there was substantial variation in the BP variable and threshold used [2]. This variability is not simply an academic problem. The selected definition determines the reported incidence of IOH, which patients are classified as exposed, and when clinicians may feel compelled to intervene.

An individualized baseline comparator is therefore appealing. A relative decrease acknowledges that the same absolute MAP may not have the same physiologic significance for every patient and is consistent with the broader concept that IOH should be personalized rather than defined exclusively from population reference values [3,4]. The approach used by Zhao et al. is an important strength of the study. Their baseline was the mean BP recorded within 24 h before surgery, which may be more representative than a single measurement obtained immediately before induction in an anxious or physiologically unstable patient.

However, an individualized threshold is only as reliable as the baseline from which it is calculated. Obtaining an accurate awake BP in infants is difficult. In a prospective study, awake values could not be obtained in 15% of infants, behavioral state influenced the measured pressure, and relative and absolute definitions of IOH were not interchangeable [3]. Even when a preoperative baseline value is available, sleep, crying, feeding, cuff size, measurement technique, concurrent respiratory support, medications, and changes in clinical condition may influence the result. Averaging measurements may reduce random variation, but it does not fully resolve these sources of bias. A precise percentage reduction calculated from an imprecise baseline may create an appearance of certainty that the underlying measurement cannot support.

The method of intraoperative BP measurement introduces an additional challenge. Zhao et al. recorded invasive BP every minute and noninvasive BP every 3–5 min, prioritizing invasive values when both were available [1]. This pragmatic approach reflects clinical care, but invasive and oscillometric measurements are not necessarily interchangeable, particularly at BP extremes. In critically ill children, automated oscillometric measurements were higher than arterial and Doppler measurements during periods of hypotension [5]. More recently, a prospective multicenter study of anesthetized children found poor agreement between invasive and noninvasive MAP, with a mean bias of 15 mmHg during hypotensive readings [6]. These studies were not limited to neonates undergoing non-cardiac surgery, but they illustrate how the measurement method itself may influence whether a patient crosses a study-defined threshold. Further, a reduction lasting more than 5 min may be characterized differently by a continuous arterial waveform than by a cuff cycling every 3–5 min.

Despite these inherent difficulties, Zhao et al. provide valuable information regarding the frequency and clinical context of neonatal IOH. The large sample size is notable for a neonatal perioperative study. Requiring both a relative decrease from baseline and a duration longer than 5 min also reduces the likelihood that a single transient or erroneous value would define the exposure. The resulting incidence of 59.8% confirms that substantial decreases in BP are common during neonatal anesthesia. Importantly, this finding should not be interpreted to mean that nearly 60% of neonates experienced inadequate organ perfusion. Rather, nearly 60% met a clearly described BP definition that can now be examined and refined in future studies.

The identified risk factors are clinically plausible and help characterize neonates who may be especially likely to develop intraoperative BP instability. Higher American Society of Anesthesiologists physical status, emergency surgery, surgery longer than 60 min, thoracic surgery, congenital heart disease, and preoperative acidosis were independently associated with IOH, whereas greater gestational age was associated with lower odds [1]. Taken together, these findings suggest that IOH is most common in neonates with less physiologic reserve and during more complex or prolonged procedures. This information may help clinicians anticipate hemodynamic instability and plan the intensity and method of monitoring before anesthesia begins.

The associations with postoperative outcomes are also important. Neonates who met the IOH definition had longer postoperative ventilation, intensive care unit stay, and hospitalization, in addition to greater rates of respiratory complications, acute kidney injury, and intraventricular hemorrhage [1]. These findings appropriately focus attention on organs that may be vulnerable to impaired oxygen delivery. However, they should be interpreted as associations rather than evidence that IOH caused the adverse outcomes. The IOH group also differed in gestational age, weight, prematurity, comorbid disease, procedure duration, hypoxemia, hypothermia, transfusion, and other factors that may independently influence postoperative recovery. IOH may be a mediator of injury, a marker of greater illness and surgical complexity, or both. The authors appropriately acknowledge the limitations of a single-center retrospective design and the need for prospective multicenter studies.

Yet the study also brings the central unanswered question into sharper focus: not simply what BP constitutes IOH, but when a measured pressure represents inadequate perfusion for a specific organ in a specific neonate. BP is primarily used as a surrogate for blood flow, and focusing on a number without considering cardiac output, vascular resistance, oxygen content, and metabolic demand may be insufficient [7]. The brain, heart, and kidneys may also have different perfusion requirements and may not become pressure-dependent at the same MAP.

Cerebral autoregulation illustrates this problem. The lower limit of autoregulation is not a fixed value shared by all patients, and available pediatric data suggest substantial interindividual variability [4,8]. In infants receiving sevoflurane anesthesia, cerebral oxygen saturation became more pressure-dependent at lower MAPs, with a more pronounced relationship below 50 mmHg [8]. These findings do not establish 50 mmHg as a universal treatment threshold, particularly in neonates, but they demonstrate why an identical BP may be tolerated by one patient and associated with impaired cerebral oxygenation in another. The individualized preoperative baseline used by Zhao et al. is therefore an important step toward personalization, but it remains a pressure-based surrogate rather than a direct assessment of cerebral, myocardial, or renal perfusion.

The absence of treatment-specific information is another important limitation. The report includes total intraoperative fluid input and transfusion, but it does not describe whether episodes of IOH were treated with fluid boluses, vasopressors, inotropes, adjustment of anesthetic depth, or other interventions. The timing, dose, and physiologic response to treatment are also not reported. Consequently, some neonates may have had brief episodes that were promptly corrected, whereas others may have remained hypotensive despite intervention. Treatment could shorten the duration of exposure, modify the association with outcome, or introduce separate risks. Without these data, the study cannot define when clinicians should intervene or which intervention is most appropriate.

Future studies should preserve the strengths of the current investigation while adding greater physiologic resolution. A standardized and reproducible preoperative baseline should be obtained when possible, and the BP device, cuff size, measurement site, and use of invasive monitoring should be recorded. Both absolute pressure and relative change from baseline should be analyzed, together with depth and duration of IOH or a time-weighted measure of BP burden. Most importantly, hemodynamic interventions and their temporal relationship to BP and outcome should be captured.

Increasingly precise BP data alone will not completely solve the problem. The next advance will require better monitoring of the end-organ perfusion that BP is intended to protect. Cerebral oximetry and autoregulation monitoring may provide information regarding the brain; measures of cardiac output and myocardial performance may better characterize systemic and coronary perfusion; and renal perfusion or early kidney-injury measures may identify risk before serum creatinine changes. None of these modalities currently provides a complete or universally validated answer for neonates under anesthesia. Nevertheless, integrating BP with organ-specific measures is more likely to identify clinically meaningful IOH than continued refinement of a single population threshold.

Zhao et al. have added valuable insight into the incidence, associated factors, and short-term outcomes of neonatal IOH. Their use of each neonate's preoperative BP as the comparator is a notable strength and is preferable to relying solely on an arbitrary definition based on age or weight. At the same time, uncertainties in the baseline measurement, differences between invasive and noninvasive monitoring, absence of treatment data, and lack of direct measures of organ perfusion limit the conclusions that can be drawn. The main message should not be that every 20% decrease in MAP requires treatment. Rather, the study demonstrates that such decreases are common and associated with important postoperative outcomes. The challenge now is to determine when a change in pressure signals compromised perfusion of the heart, brain, or kidneys. Better end-organ monitoring, rather than another universal BP number, will be necessary to answer that question.

## Conflicts of interest

The author declares no conflicts of interest.

## Data Availability

N/A.
